# Rebuilding child health in South Kivu, Democratic Republic of Congo (DRC): evaluating the Asili social enterprise program

**DOI:** 10.1186/s13031-022-00454-0

**Published:** 2022-05-07

**Authors:** Rasika Behl, Sofia Ali, Jonathan Altamirano, Abraham Leno, Yvonne Maldonado, Clea Sarnquist

**Affiliations:** 1 Department of Pediatrics, Stanford University School of Medicine, 453 Quarry Rd., Palo Alto, CA 94304 USA; 2 Eastern Congo Initiative, Bukavu, South Kivu Democratic Republic of Congo

**Keywords:** Social enterprise, Armed conflict, Market segmentation, Democratic Republic of Congo, Child health

## Abstract

**Background:**

The Democratic Republic of Congo (DRC) has a long history of conflict and ongoing local instability; the eastern provinces, including South Kivu, have been especially affected. Health systems and livelihoods have been undermined, contributing to massive inequities in access to health services and high rates of internal displacement. Asili, an innovative social enterprise program, aimed to provide essential community services and improve the health of under-five children in two South Kivu communities, Mudaka and Panzi, via provision of small-format, franchisable health clinics and clean water services.

**Methods:**

We evaluated utilization and acceptance of Asili services in two study sites, Mudaka and Panzi. Data collected included questions on housing conditions, food security, and at follow up, Asili membership and use, satisfaction with services, and recommendations for improvement. Structured pre- and post-interviews with primary caregivers of families with under-five children were the primary source of data with additional community input collected through focus group discussions.

**Results:**

At baseline, we enrolled 843 households in Mudaka and 890 in Panzi. Market segmentation analysis illuminated service usage patterns, showing Asili services were well received overall in both Mudaka and Panzi. Families reporting higher levels of proxy measures of socioeconomic status (SES), such as electricity, land ownership, and education, were more likely to use Asili services, findings that were further supported by focus group discussions among community members.

**Conclusions:**

Rebuilding health infrastructure in post-conflict settings, especially those that continue to be conflict-affected and very low SES, is a challenging prospect. Focus group results for this study highlighted the positive community response to Asili, while also underscoring challenges related to cost of services. Programs may need, in particular, to have different levels of costs for different SES groups. Additionally, longer follow-up periods and increased stability may be needed to assess the potential of social enterprise interventions such as Asili to improve health outcomes, especially in children.

***Trial registration*:**

Institutional Review Board approval for this study was obtained at Stanford University (IRB 35216) and the University of Kinshasa, DRC. Further, this study has been registered on Clinicaltrials.gov (record NCT03536286), retrospectively registered as of 4/23/2018.

## Introduction

Over the last several decades, the Democratic Republic of Congo (DRC) has endured ongoing armed conflict, leading to widespread destruction of lives and livelihoods. The health care system has struggled to provide basic care to citizens, and in 2018, an estimated 72% of the population lived below the global poverty line [1, 2]. The eastern provinces, including South Kivu, have been especially affected by local instability and its negative impacts on health and economic wellbeing [3]. This ongoing instability has also led to high rates of displacement, including over 2.2 million new internally displaced people in 2020 [4]. A large Ebola outbreak began in 2018 and claimed the lives of 2299 people, causing further destabilization and displacement [5].

In local communities across DRC, especially the eastern provinces, many families have unmet needs, including access to essential resources such as clean water and food. Recent data indicate that less than half of households live near an improved source of drinking water [6] and under-five mortality in DRC is ninth highest in the world, with nearly one out of every ten children dying before reaching age five [7]. Asili, meaning ‘foundation’ in Kiswahili, is an innovative initiative aiming to address these community needs, with a focus on under-five child health, by integrating existing, previously independently tested social enterprises into a uniform platform.

Social enterprises aim to address social inequities or vulnerabilities within communities, utilizing commercial strategies to simultaneously generate income and profit while benefiting society and the environment [8]. These businesses operate outside of traditional systems focused solely on promoting welfare but share the goal of alleviating social inequities. In their conceptual model, Roy et al. describe social enterprises as business entities with a social mission that directly, through trading or service delivery, or indirectly, through investment of profits from trading activity, intervene at the individual- and/or community-level to improve health and wellbeing [9]. Haugh outlines six stages of “social venture” development: opportunity identification, idea articulation, idea ownership, stakeholder mobilization, opportunity exploitation, and stakeholder reflection [10]. Across these stages, social enterprises build on existing resources within the community, promote sustainability through direct involvement of local stakeholders, and fill demand unmet by the public sector, which increases their potential to improve health and wellbeing [8, 9].

Evidence of the impact of social enterprises on health outcomes, especially in conflict-affected areas, is minimal. However, some studies have examined social enterprise and their impact on under-five children. An example of a promising intervention is the Angola Social Action Fund (ASAF), which finances income-generating activities and local-level projects in health education, water, and sanitation. A study of ASAF found statistically significant decreases in rates of stunting in ASAF communities compared to non-ASAF communities, suggesting that social enterprise interventions may improve child health [11]. In Kenya, an example of a franchise clinic model is The HealthStore Foundation’s (HSF’s) CFWShops, which train local nurses to own and run child and family wellness clinics with guidance and support from HSF. Under-five children living in closer proximity to the CFWShops received more total vaccinations than those who lived farther; additionally, acutely ill children living in proximity to CFWShops had higher probability of receiving any medical treatment. These findings indicate that the franchise clinic model may improve access to preventive care for under-five children living in low-resource settings [12]. There may be even greater potential for improvement in conflict-affected regions, where access to services may be especially poor; however, there is little current research on social enterprises in these settings.

Asili was developed using a human-centered design (HCD) approach undertaken by IDEO.org in partnership with the American Refugee Committee (ARC) in South Kivu, DRC [13, 14]. The HCD process was used to identify essential needs for the local community and prototype community-supported solutions to meet those needs. Based on the results of this process, the final Asili enterprise included small primary care health clinics and clean water distribution systems that were designed to eventually become self-sustaining, franchisable social enterprises. The current study aimed to assess utilization of and community response to Asili services in South Kivu as well as address the gap in understanding how social enterprises function in conflict-affected regions.

## Methods

### Intervention

ARC and IDEO.org used an HCD approach to design the Asili intervention. Asili services were available to everyone in the communities of Mudaka and Panzi. Community members could access Asili in one of two ways: (1) by paying for a monthly membership (defined as an Asili member) or (2) by purchasing services individually (defined as an Asili user). Asili members received five 19-L jerry cans of water per day and subsidized prices for clinic appointments and basic medicines. Asili users paid higher fees for individual jerry cans of water and clinic services and medicine; however, access to Asili services was not otherwise restricted. The goal in charging for Asili services and memberships was to eventually develop self-sustaining services that would create jobs through social franchise opportunities, thus rebuilding livelihoods within the community whie also providing needed services.

### Study setting

There were two primary study sites, Mudaka and Panzi, both in Bukavu, South Kivu, DRC. Catchment areas for both study sites were determined by the local ARC team and were subsequently mapped using GPS ‘clickers’ and Google Earth. Mudaka was divided into six regions that were defined according to population distribution and geographic factors (“close to the market”, “close to the lake”, etc.), in order to accurately represent the local population. Panzi was divided into four regions, with three regions centered around planned Asili water points, and one centered around the planned Asili clinic.

Community-level consent was obtained from the Mwami (King) of Kabare, after which data collectors were sent door-to-door to identify households with under-five children and obtain individual consent from adults ages 18–65 to participate in the study. Data collectors included all mothers with under-five children in the household in the study because some households are multi-family, for a variety of reasons. Households were sampled by picking every third or fourth household to evenly represent all areas of each site; however, populations of under-five children varied highly from region to region.

### Data collection

Study data were primarily collected through household surveys administered to heads of household in families with under-five children; additional community input was collected through small focus groups during follow-up. Experienced field staff were hired from local communities and overseen by DRC-based data collection firms. Local staff helped with translation of surveys and focus group data and ensured cultural appropriateness. Before each round of data collection, the local team and university team researchers met for three days to practice surveys and review the protocol and ethics. All data collectors were fluent in French; most also spoke fluent Kiswahili and/or Mashi. Data was collected via surveys deployed on Nexus 7 tablets using the Research Electronic Data Capture (REDCap) mobile application. REDCap is a secure, web-based software platform designed to support data capture for research studies, providing (1) an intuitive interface for validated data capture; (2) audit trails for tracking data manipulation and export procedures; (3) automated export procedures for seamless data downloads to common statistical packages; and (4) procedures for data integration and interoperability with external source [15, 16].

**Fig. 1:**
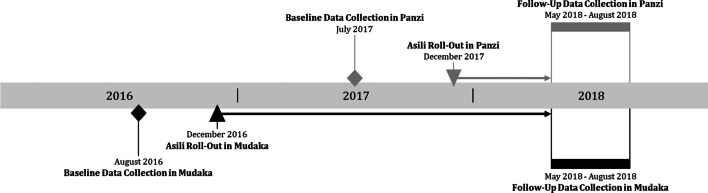
Timeline of data collection in Mudaka and Panzi

In Mudaka, baseline data were collected over several weeks in August 2016, and Asili services were rolled-out in December 2016. In Panzi, baseline data were collected in July 2017, and services were rolled-out in December 2017. Follow-up data in Mudaka and Panzi were collected between May and August 2018 to maximize follow-up time periods before the end of the study in mid-2018 (Fig. 1).

### Outcomes

Data collected from households included demographics of primary caregivers and children under-five, housing conditions, food security, and access to water, plumbing, and electricity. At follow-up, additional questions were asked about Asili membership, utilization and satisfaction with Asili services, and recommendations for improvement.

Survey instruments primarily relied on validated questions from Demographic and Health Survey (DHS) modules for DRC [17]. These modules were pretested in villages in the Bukavu area not participating in the study to assure appropriateness for this population. At baseline and follow-up, interviews were conducted in French, with occasional translation into Kiswahili and Mashi for better comprehension, as needed. Institutional Review Board approval was obtained from both universities.

Additional outcomes of this study included measures of disease incidence, as a proxy measure of child mortality. Maternally-reported diarrhea, cough, and fever in the preceding 14 days were used to represent incidence of diarrheal disease, pneumonia, and malaria, respectively, as the leading causes of under-five child mortality in DRC [18]. These proxy measures of child mortality and under-five health were originally intended as the primary outcome of our study, using a combination of self-reported data from primary caregivers and medical chart data. However, due to numerous unanticipated challenges obtaining chart data as well as difficulties with maternal recall in large and busy households, data quality was so poor as to warrant non-inclusion in these findings.

### Data analysis

#### Descriptive analysis

Data were stratified by Asili membership (member vs. non-member) and usership (user vs. non-user) during analysis in Mudaka and Panzi. Study sites were analyzed separately, for several reasons: Asili roll-out occurred in Mudaka a full year before roll-out in Panzi, Panzi is more urban than Mudaka, and the populations of the two areas are heterogenous, all making comparison not especially useful in terms of understanding the social enterprise model in this setting.

#### Market segmentation analysis

Market segmentation analysis can be used to observe trends in uptake of a product or intervention, such as Asili. A form of exploratory data analysis, market segmentation uses algorithms to divide populations into distinct subgroups with common characteristics that can be described in order to understand use of a product or service [19, 20]. A common method to create these groups is through k-means clustering, a form of exploratory unsupervised learning that uses an iterative algorithm to create distinct clusters based on variables of interest. These clusters can then be described in order to characterize the subgroups that compose the initial population.

In Mudaka and Panzi, k-means clustering used data collected at baseline to create consumer profiles within these communities and assess which profiles were most likely to access Asili services. Land and animal ownership, electricity, type of toilet, and self-reported food scarcity were machine-selected to create the clusters for both study sites, with Mudaka additionally using drinking water and roofing material in its algorithm, while Panzi additionally used flooring material.

Clusters were organized by increasing proportion of households reporting use of Asili services, and trends across the different demographic variables were observed. All of these characteristics, except for food insecurity, are proxy indicators of wealth used in the DHS wealth index to measure economic status [21]. Thus, we interpreted trends across these variables as reflective of differences in Asili usage by household wealth. Households with missing cluster variables or unknown village data at baseline were excluded from the analysis. Variables without notable trends across clusters, including drinking water and roofing material for Mudaka and type of toilet for Mudaka and Panzi were not reported.

## Results

### Mudaka

At baseline, a total of 843 households, representing 845 mothers and 1,328 under-five children, were surveyed in Mudaka. Average household size was 6.88 people, and almost a third (31%; n = 259) of mothers had less than one year of formal education, with only 22% (n = 184) having secondary education. At follow-up, 287 households, 288 mothers and 359 under-five children were surveyed, with similar average household sizes (6.90); 6% of households were Asili member families, and 46% (n = 133) were Asili user families. Overall household retention in Mudaka was 34% (287/843); local instability and widespread internal displacement contributed to difficulties in locating families at follow-up.

**Fig. 2:**
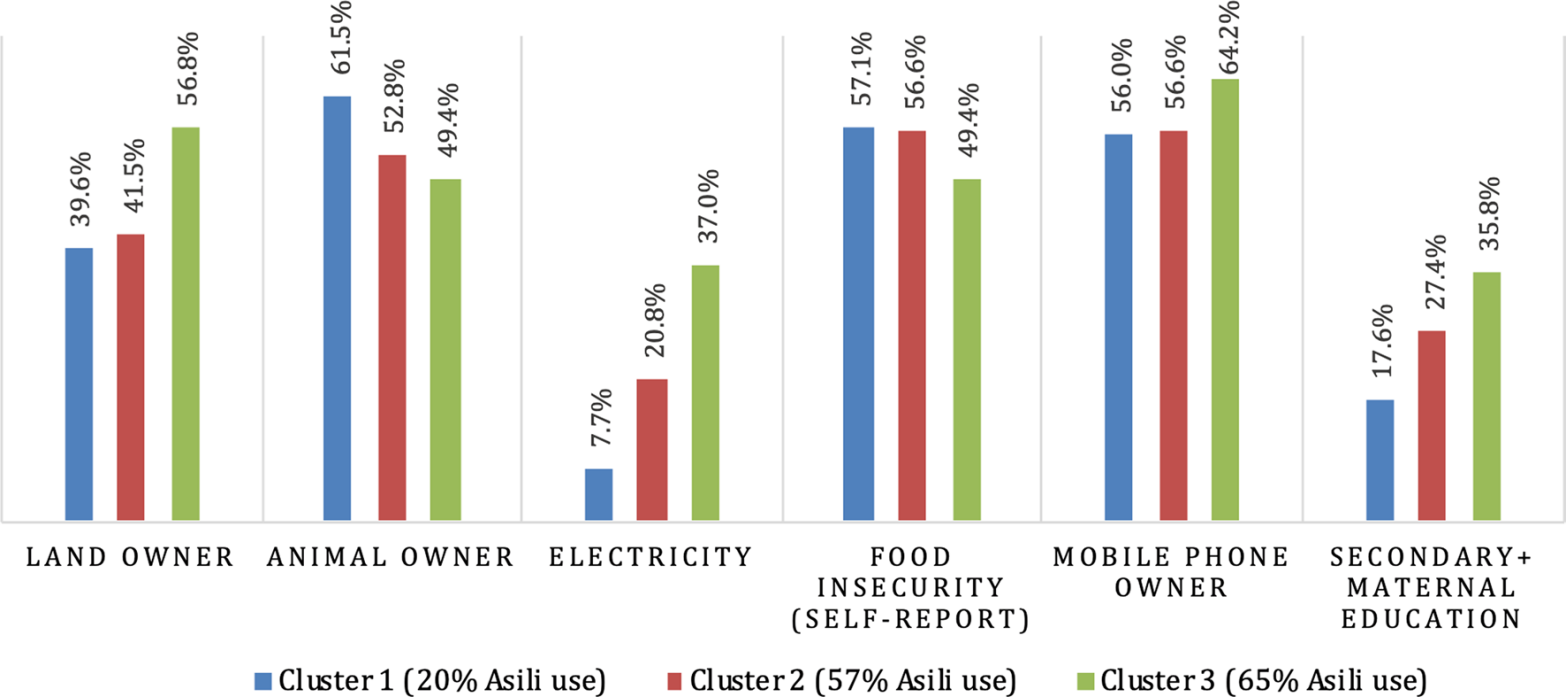
Market segmentation analysis; Mudaka*. *Data reported is percentages of households answering ‘yes’ to variables above, except maternal education, which was dichotomized to ‘any primary’ and ‘secondary or above’

Households in Mudaka were segmented into three clusters (Fig. 2) by market segmentation analysis. All of the clusters contained households from different parts of Mudaka. Asili use ranged from 20% of households in Cluster 1 to 65% of households in Cluster 3. Asili membership was less frequent than Asili use across all clusters but did show a similar trend, as Clusters 2 and 3 reported higher proportions of Asili membership compared to Cluster 1, at 8% and 6% respectively.

Electricity, land ownership, mobile phone ownership, and maternal education were highest in Cluster 3 and lowest in Cluster 1. Comparing households in Cluster 3 to Cluster 1 across these variables, electricity was almost five times as prevalent (37% vs 8%), land ownership was almost 20% higher (57% vs 40%), mobile phone ownership was almost 10% higher (56% vs 64%), and the proportion of mothers who completed secondary education or higher was double (36% vs 18%).

Animal ownership, which was not stratified in the data, and food insecurity were highest in Cluster 1, at 62% and 57% respectively. Clusters 2 and 3 showed lower proportions of both, with animal ownership at 53% for Cluster 2 and 49% for Cluster 3, while food insecurity was reported at 56.6% in Cluster 2 and 49% in Cluster 3.

The trends in these proxy measures of wealth suggest that Asili use decreases with declining wealth. This finding was reflected by participants in focus group discussions in Mudaka:

The biggest challenge in accessing Asili's services is the high price at its clinic and its standpipes.

While many participants expressed concerns about the high cost of using Asili’s services, some of them viewed this cost as marginal compared to the benefit of having healthier children:

…we cannot benefit from its [Asili’s] services without having money, but I emphasize that it has improved the health of my children because my children do not get as sick as before thanks to access to clean water and its cost, I do not spend a lot of money on care.

### Panzi

In Panzi, a total of 890 households, with 892 mothers and 1,459 under-five children, were surveyed at baseline. An average of 7.04 people lived in each household; almost a third of mothers (29%; n = 256) completed primary school and almost half (44%; n = 387) completed secondary education. During follow-up data collection, a total of 513 households, representing 513 mothers and 832 under-five children, were surveyed; 1% of households were Asili members, and 43% reported using Asili services. Average household size at follow-up, 7.21 people, was similar to baseline. Overall household retention in Panzi was 58% (513/890) for similar reasons as in Mudaka, though Panzi’s more urban location and shorter time period between enrollment and follow-up likely allowed for higher retention.

**Fig. 3:**
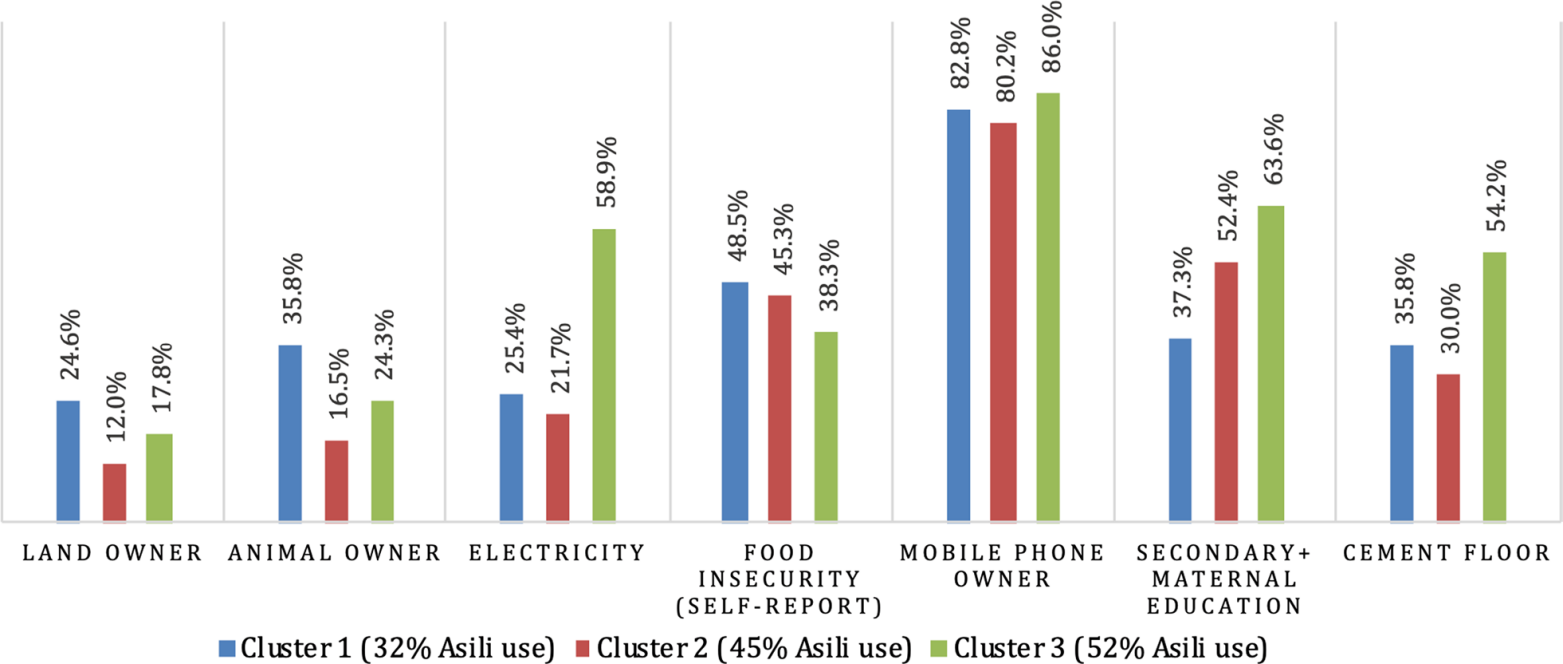
Market segmentation analysis; Panzi*. *Data reported is percentages of households answering ‘yes’ to variables above, except maternal education, which was dichotomized to ‘any primary’ and ‘secondary or above’ and floor material, dichotomized to ‘dirt’ and ‘cement’

In market segmentation analysis, households in Panzi were divided into three clusters (Fig. 3). Asili use ranged from 32% in Cluster 1 to 52% in Cluster 3. Asili membership in Panzi was less common than use; only 1–2% of any cluster were members. All three clusters contained households from different areas.

In Cluster 3, 64% of mothers in Cluster 3 reported having secondary or post-secondary education. In comparison, in Cluster 1, 25% of households had electricity, 83% of households had a mobile phone, and only 37% of mothers had secondary or post-secondary education.

Land and animal ownership, as well as food insecurity, were all highest in Cluster 1, at 25%, 36%, and 49% respectively. Cluster 3 had lower levels of all three variables, with land and animal ownership at 18% and 24% respectively and food insecurity at 38%, the lowest level across the three clusters. By contrast, electricity and mobile phone ownership were highest in Cluster 3, at 59% and 86% respectively.

Finally, flooring material varied by cluster. In Cluster 1, 58% of households had dirt floors, and 36% had cement floors. In Cluster 3, these proportions were inverted, with 54% of households having cement floors, and 37% of households having dirt floors.

As in Mudaka, trends across these characteristics—for example, highest proportion of electricity use, mobile phone ownership, and cement flooring in Cluster 3—indicate that use of Asili services in Panzi is associated with higher levels of wealth. One focus group participant asserted:

We did not use its services because of the lack of money, but if we had money, its services will be used without problem.

A second respondent stated:

The price of water remains the biggest challenge that drives us to not access Asili's services.

As in Mudaka, some survey respondents explained that although the cost of accessing services was high, they may save money overall due to the high quality of care provided at Asili clinics:

…Before when children were sick, we brought them in unreliable health structures and which did not give them good medications, and this led to relapses and so parents had to spend at all times. But with the arrival of Asili the child gets sick and he goes to Asili for healing only once.

## Discussion

Social enterprise interventions aimed at meeting community needs while allowing for income generation have shown some promise in low and middle-income country settings. Market segmentation analysis, along with low uptake numbers for Asili membership, suggest that the price point of the services, while low in absolute terms, was still out of reach of many families in this high-poverty, conflict-affected region. Nonetheless, those families who did access Asili services appreciated the quality and believe that the return on investment was high.

Challenges with creating price-points for basic services that support eventual self-sustenance but are still within economic reach of the target population have been encountered in many previous studies [22–24]. This issue may be especially pronounced in communities with a long history of receiving free services, such as conflict-affected and fragile areas [23, 25]. Although Asili employed an HCD process in its inception, it may be useful for program implementers to employ HCD directly for the price-point question. Another strategy may be to integrate cost–benefit analyses and social return on investment (SROI) analyses as part of future evaluations, to allow for comparisons of the monetary cost with the social benefits of achieving important health outcomes [26]. This could shift the programmatic focus from making the intervention entirely self-sustaining and allow for more varied and realistic measures of success in a challenging setting, though self-sustaining income generation can remain a longer-term goal for the program.

A noteworthy finding, which may be useful in designing future outreach for Asili, was that household clusters with higher maternal education reported higher Asili service utilization. Prior research has found that educated mothers are more likely to understand and utilize disease-prevention measures, such as antenatal and maternal care and routine immunization [27–29]. These women are also more likely to access health services for a sick child, to follow treatment instructions, and to set clean water and sanitation as household priorities [30]. Mothers who are more educated are also more likely to belong to wealthier families [31], indicating that the association with increased access to Asili could be the result of a combination of wealth and education. One option for increasing use of and membership in Asili may be to utilize these member mothers as peer influencers or community health outreach workers. These types of peer-to-peer models have shown success in other settings, including Living Goods in Uganda, the Accredited Social Health Activist (ASHA) model in India, the Lady Health Worker (LHW) model in Pakistan, and the Health Extension Worker (HEW) program in Ethiopia [32]. The ASHA, LHW, and HEW programs were successful at rapid achievement of scale at the national level but face challenges around sustainability. The Living Goods model has shown even more promise, with an under-five child mortality reduction of 27% after 3 years [33].

### Limitations

Key limitations of this study included: a low retention rate and short follow-up period, self-report of symptoms related to outcomes, and confusion around definitions of ‘Asili membership’.

Several factors affected our ability to retain families in this study. Although we collected GPS coordinates, address ‘descriptions’, and phone numbers for every household, high mobility and lack of formal addresses were a challenge, and many families were not traceable at follow-up. However, no statistically significant differences on the disease symptom outcomes between those followed and those lost suggest that the high loss-to-follow-up may not have greatly affected the results (data not shown). Follow-up time was also a limitation, especially in Panzi, where the lack of an effect may have been primarily due to a short follow-up. This was due to longer-than-expected service roll-out times, which were beyond the control of the research team.

Self-report of symptoms was also a limitation, as heads of household (usually the family matriarch) did not always recall if a child had been ill in the previous 2 weeks and, in particular, if a single child in a large household had been mildly or moderately ill compared to a child with severe symptoms. This, alongside numerous unanticipated challenges that prevented us from accessing supplemental chart data and this, alongside well-known limitations of relying only on self-reported data, impacted our ability to use child health outcome data.

A final key limitation was that the concept of ‘membership’ in Asili was not well understood by the study population. While Asili services were available to non-members and members, data collectors anecdotally reported that respondents were confused about the difference between ‘user’ and ‘member’. As such, we may have missed members who identified as users, and therefore underestimated Asili membership and potential effects of the intervention.

## Conclusion

Our analysis found that Asili was largely well-received, if not always affordable by two communities in South Kivu, DRC. Qualitative data suggest that community members see the potential value of Asili, especially for the health of their children. Refining of the intervention and its pricing, along with cost-benefit analyses on a more mature version of Asili with longer-term follow-up, is warranted.

## Data Availability

The datasets generated and/or analyzed during the current study are not publicly available due participant confidentiality but de-identified data can be made available from the corresponding author on reasonable request.

## Abbreviations

DRC: Democratic Republic of Congo
SES: Socioeconomic status
HCD: Human-centered design
ARC: American Refugee Committee
ASAF: Angola Social Action Fund
HSF: HealthStore Foundation
GPS: Global Positioning System
REDCap: Research Electronic Data Capture
DHS: Demographic and Health Survey
SROI: Social Return on Investment
ASHA: Accredited Social Health Activist
LHW: Lady Health Worker
HEW: Health Extension Worker
